# Evaluating the effect of environmental conditions on high compressive strain rates in unfilled and filled neoprene rubbers

**DOI:** 10.1177/00952443231197727

**Published:** 2023-08-22

**Authors:** Elli Gkouti, Muhammad Salman Chaudhry, Burak Yenigun, Aleksander Czekanski

**Affiliations:** Department of Mechanical Engineering, Lassonde School of Engineering, 7991 York University, Toronto, ON, Canada

**Keywords:** Filled neoprene rubber, high strain rate, temperature effect, humidity effect, Kolsky bar

## Abstract

Elastomers are known for their strain-rate-dependent properties not only to quasistatic but also to high strain rate deformations, where mechanical behavior is significantly affected by inertia. Concurrently, environmental changes, such as temperature and humidity variations, can impact their stress response to deformation. This study investigates the effects of material layers within neoprene samples on mitigating these environmental changes. While the presence of an intermediate layer proves effective against temperature and humidity influence, it fails to block the impact of increasing high strain rates. Moreover, the different humidity levels at room and elevated temperatures do not significantly alter the mechanical behavior of filled neoprene samples compared to pure neoprene. Notably, in unfilled neoprene, an increase in humidity levels, other than an absolutely dry environment, leads to a notable stress level rise at room temperature, while under elevated temperature conditions, there is a significant stress decrease with increasing humidity. However, neoprene filled with polyester/cotton or nylon displays resilience to diminishing mechanical behavior under various temperature and humidity regulations, indicating that the material layer within these samples effectively “protects” the rubbers from potential stress lapses observed in unfilled neoprene. While a high strain rate compression affects the behavior of the filled variants significantly, increasing humidity and temperature have minimal impact on their stress levels. These findings offer valuable insights into the dynamic responses of elastomers to environmental changes, highlighting the advantages of using filled rubbers in diverse applications.

## Introduction

Neoprene (or polychloroprene) rubber has been widely used in many applications due to its chemical stability and unique properties such as temperature, oil, and abrasion resistance.^1^ Hence, it can be adopted in a wide application range relative to engineering, automotive, coating, and aquatics.^2,3^ However, the latest application innovation requires even more enhanced durability and stiffness of rubbers. Thus, the filled rubbers were fabricated to satisfy those requirements.^4–7^ In the case of neoprene, a common embedding reinforcement is single or multiple material layers in their composition. Depending on the application, these layers might be thick fabric or even nylon layers. Cotton fabric- and nylon-reinforced neoprene samples were selected for our investigation, which are widely used for applications related to flange gaskets, diaphragms, packing, and in any field where high strain rate compression might squeeze nonreinforced neoprene rubber out of place.

Although most applications require exposing materials to high-speed deformation, rubbers are extremely sensitive to strain rate due to unpredictable inertia effects.^8–10^ It has already been explored, from several existing studies found in the bibliography, that the high strain rate impacts elastomers, and it has been proved that the required stress at a given strain value gradually elevates with increasing strain rate.^11–14^ This increase in stress with strain rate is due to the inability of sub-molecular chain motions (e.g., rotations) to occur within the experiment's duration. The low strain rate limit stress is that of viscous flow, and the high strain rate limit stress arises from the maximum instantaneous stress when there has been no time for any viscous effect.^9,15^

In order to record elastomers' response to high strain rates, a dynamic MTS or Kolsky bar equipment can be used, as they can function in high-speed deformation.^16–18^ During a Kolsky bar test, the specimen is sandwiched between two slender rods, the input and output bars, or incident and transmitted bars, which are instrumented with strain gauges.^19–21^ A loading system, typically a gas gun, propels a shorter third rod or striker into the incident bar. This generated a stress wave, the incident signal, propagating down the bar to the specimen. At the bar-specimen interface, the change in impedance (density times sound speed) between the bar and the sample causes some of the wave to be reflected down the input bar and some to be transmitted to the output bar, forming the reflected and transmitted signals, respectively. Typically, all three waves are measured using strain gages mounted on the incident and transmitted bars. It is common practice to mount two strain gauges at each location that is opposed to each other, which will result in canceling out bending waves and, subsequently, measurement of a purely compressive pulse.^22^

In real-life applications, it is evident that deformation in any strain rate range may occur under various environmental conditions. For this reason, it is necessary to study the mechanical behaviour of these materials over different conditions. Hence, accounting for temperature and humidity regulations is mandatory when rubber's response at high strain rates is being investigated.^12,23–25^ Besides, nonreinforced elastomers are highly impacted by excessive temperature changes that might contribute to the structure's overall degradation. Since reinforcing rubbers with extra intermediate material layers enhances their stiffness and duration, following this pattern will improve their resistance to a combination of humidity and temperature regulations. Until now, most experimental studies have primarily focused on examining the effects of temperature on rubbers. However, depending on its composition, silicone can stand out due to its remarkable weatherability. Even when exposed to moisture conditions, it was observed to absorb only 1% of moisture, maintaining its mechanical strength and electrical properties even after prolonged exposure to water.^26^ This exceptional attribute is further enhanced in filled silicone. In contrast, neoprene rubber experiences a negative impact on its mechanical properties during both static and dynamic deformation, as it shows a reduction in stress with increasing temperature.^2,27^

The current study aims to investigate filled and unfilled neoprene’s high strain rate response to environmental changes when regulating the conditions by increasing and removing the moister at different temperatures. Two types of filled neoprene were selected, with the reinforcement layer being either cotton fabric or nylon. To analyze the behaviour of filled rubbers compared to nonreinforced neoprene, we selected to perform high strain rate compression tests for absolutely dry conditions by removing humidity from the room temperature environment. In addition, the moister condition at 23°C temperature was investigated by increasing real humidity (RH) to 50%. We repeated the same experimental procedure to simulate real applications' conditions by adding another variable affecting neoprene's behaviour. Thus, we followed the same setup for the elevated temperature of 60°C. All testing results prove that an intermediate material layer in the rubber enhances its durability and highly resists environmental changes. Depending on the filament's material, the stiffness of rubber can be significantly improved.

## Methodology

### Materials and samples’ geometry

For the current paper, neoprene was used for high strain rate compression with or without being reinforced with an intermediate layer. Assuming that the mechanical behaviour will differ depending on the filament's material, we selected two types of reinforced neoprene, one with a cotton fabric and another with a nylon layer. Specifically, a layer of nylon or cotton fabric was embedded in the middle of each sample sheet.

The material used in this investigation was a commercial neoprene rubber supplied by McMaster-Carr, Canada, as a 1/8-inch-thick sheet with 1300 kg/m^3^ density. For the series of compression experiments, suitable circular samples with a diameter of 13.9 mm were carefully machined out from the sheet, ensuring no substantial temperature increase. Although one layer of the filler material (cotton or nylon) was embedded in the intermediate of the rubber sheet, the sample dimensions of filled neoprene are the same as the unfilled rubber.

For all experimental cases, we have results for a minimum of three samples. In cases where the mean error exceeded 5%, an additional test was conducted subsequent to result evaluation. Consequently, the obtained data from at least three samples in each experimental case were utilized to generate the average graphs shown in the following figures.

### Experimental setup

#### High strain rate compression-Kolsky bar

The design and procedure for the Kolsky bar equipment for soft materials testing are already analyzed in several studies.^12,19^ In the current investigation, an in-house split-Hopkinson (Kolsky) pressure bar was used for the compression experiments at high strain rates.^16^ Specifically, Ti-6A1-4V alloy bars with a diameter of 12.7 mm were used. Although the bar material could be changed to provide a lower impedance mismatch with neoprene, the data collected using titanium alloy bars were of sufficient quality for the study presented in this paper. The striker bar can reach speeds around 20 m/s, corresponding to an average strain rate of around 4 × 10^3^ s^−1^. Moreover, we used petroleum jelly as a lubricant for all experiments to avoid frictional effects influencing the mechanical response.

**Figure 1.**
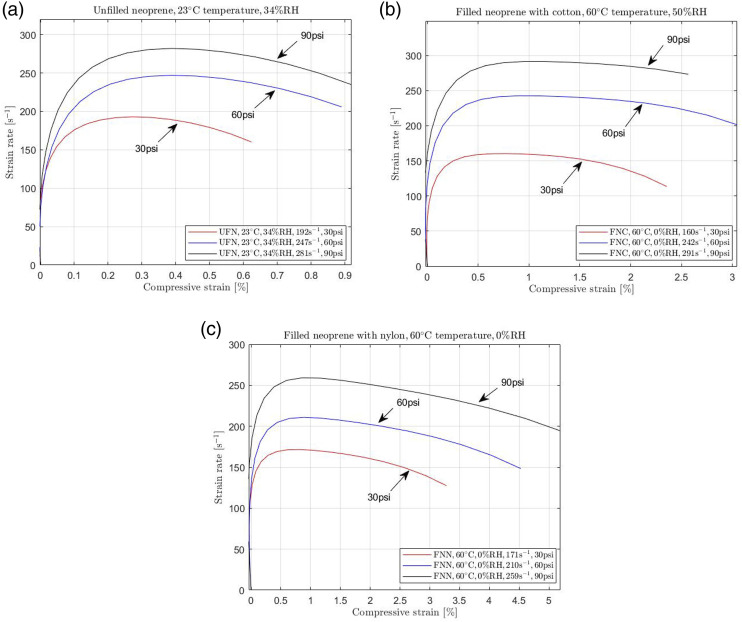
Strain rate achieved for different pressures in a Kolsky bar equipment for (a) unfilled neoprene (UFN) at 23°C temperature, 34% RH (ambient conditions), (b) for filled neoprene with cotton fabric (FNC) at 60°C temperature, 50% RH, and (c) for filled neoprene with nylon (FNN) at 60°C temperature, 0% RH.

Although the experimental investigation was performed for a high strain rate range, we tested all samples by applying three different pressures, resulting in different strain rates. Specifically, we loaded at 30 psi, 60 psi, and 90 psi pressure for all cases. However, the resulting high strain rate differs due to environmental changes and equipment variations. Due to the equipment's function, based on the pulse propagation, the strain rate is not constant throughout the experiment. However, after a short period, it approximately reaches specific values, which provides accurate results for the mechanical behaviour of rubbers. Since the Kolsky bar experiment relies on one-dimensional elastic wave propagation in metallic bars, the procedure involves releasing pressurized gas at different pressures (in psi) to accelerate the projectile. This operation impacts the bar holding the elastomer sample, inducing loading. Different strain rates can be achieved in the samples by adjusting the impacting velocities. Precise calibration is indispensable for establishing a correlation between effective strain rate and pressure settings to ensure successful dynamic tests on elastomers. Specifically, this calibration is crucial to ensure a uniform strain rate region during loading. As elastomer responses depend on the strain rate, comparing samples exposed to the same strain rate profile becomes essential to obtain meaningful results. Figures 1(a), 1(b) and 1(c) show the loading profiles for different conditions and neoprene samples.

Figure 1(a) shows the different strain rates of unfilled neoprene at 23°C, provided by applying different pressures. Namely, applying 30 psi pressure for compressing the sample in the Kolsky bar equipment, the strain rate became constant to the approximate value 192 s^−1^. Furthermore, for 60psi and 90psi, the strain rate became constant to the approximate values 247 s^−1^ and 281 s^−1^, respectively. Figures 1(b) and 1(c) show similar results for filled neoprene for different humidity levels at 60°C temperature. All strain rate levels became almost constant depending on the experimental conditions after a specific period. As shown in Figure 1, the strain rate is constant after reaching nearly 0.2% strain for both filled and unfilled neoprene. Similar results are recorded for all conditions and rubbers used in the current study.

#### Different environmental conditions

It is widely explored that neoprene's mechanical behaviour degrades after exposure to a higher temperature than the ambient. Nevertheless, the experiments' humidity conditions are often constrained to a specific level. In our study, the ambient conditions were measured at 23°C with 34% RH. When increasing the temperature to 60°C using the Thermostream equipment, the environment was recorded to be absolutely dry (0% RH). To analyze the humidity effect on neoprene rubber, we had to regulate the environmental conditions accordingly to gain comparable variables. Hence, we performed high strain rate compression tests at 23°C and 60°C temperatures. To investigate the environmental impact on this rubber – filled and unfilled - we regulated real humidity in three levels: 0% RH providing absolute dry conditions, 34% RH comparing its behaviour to the ambient with the corresponding response at elevated temperature, and finally at the increased humidity level of 50% RH for investigating considerable moister conditions at both temperatures.

It must be mentioned that all groups of experiments aim to show the different stress-strain responses between unfilled neoprene and neoprene reinforced with either cotton fabric or nylon layers. Since our study required additional testing at various temperature and humidity conditions, experiments were performed in other than the ambient environment. To ensure equilibrium, all coupons were placed in a chamber controlling temperature and humidity for at least 2 hours in the desired environment before the experiment began. When the samples were ready for testing, each was placed (sandwiched) between the two rods of the Kolsky bar for high-strain rate compression tests.

## Results and discussion

### Nonreinforced neoprene

**Figure 2.**
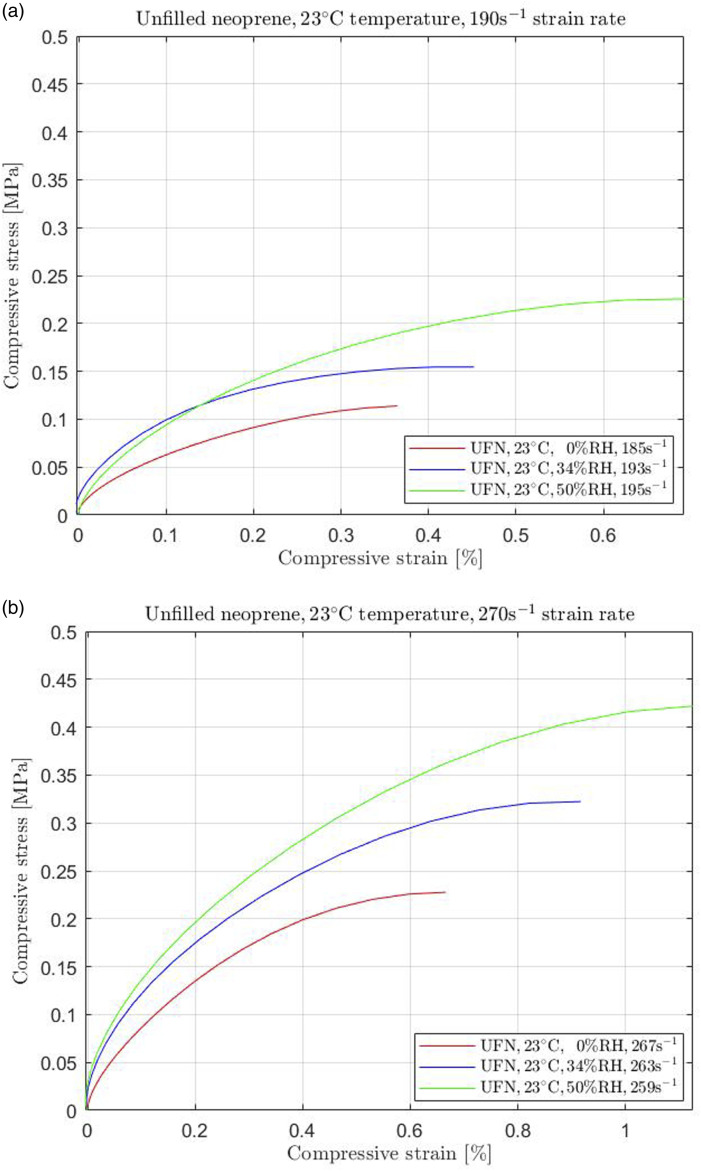
Unfilled neoprene (UFN) subjected to (a) 190 s^−1^ and (b) 270 s^−1^ high strain rate compression at 23°C temperature for 0% RH, 34% RH, and 50% RH.

**Figure 3.**
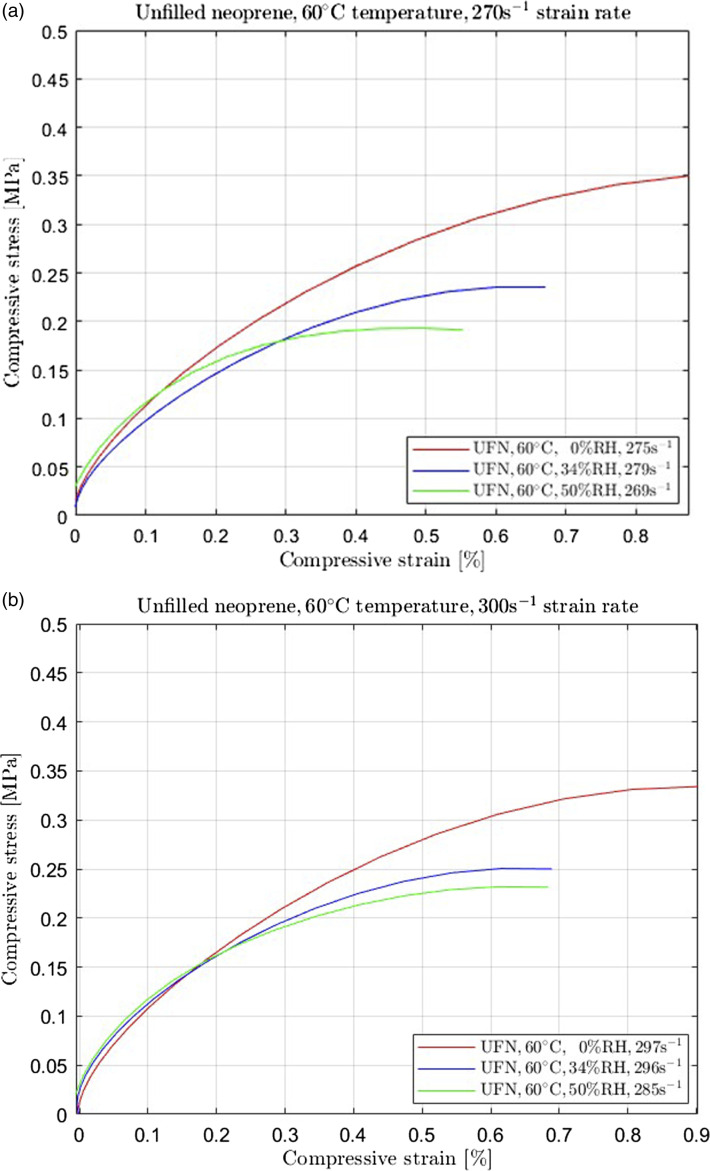
Unfilled neoprene (UFN) subjected (a) 270 s^−1^ and (b) 300 s^−1^ high strain rate compression at 60°C temperature for 0% RH, 34% RH, and 50% RH.

Changing the environmental conditions when unfilled neoprene is subjected to high strain rate compression significantly impacts its mechanical behaviour, as observed in Figures 2, 3. Table 1 displays the average percentage approximations of stress changes at different high strain rates compared to reference conditions (labeled REF) for each temperature. The reference conditions for both temperatures were set to the absolute dry environment (0% RH). Consequently, the changes are presented as percentages of increase or decrease (indicated by a minus symbol) in terms of the reference conditions of each material.

**Table 1.** Variations in peak strength of unfilled Neoprene UFN, Filled Neoprene With cotton (FFC), and Filled Neoprene With Nylon (FFN) Under different Temperature and Humidity Conditions. The Percentage Values Represent the Average Change in Stress for Both Strain Rate Cases, With REF Denoting the reference Conditions Used to Calculate the Percentage Changes.

Temperature	23°C			60°C		
Humidity	0 %RH	34 %RH	50 %RH	0 %RH	34 %RH	60 %RH
UFN	REF	35.8%	61.7%	REF	−16.2%	−25.8%
FFC	REF	4.1%	2.9%	REF	−1.0%	−1.5%
FFN	REF	0.6%	2.0%	REF	−0.1%	−0.8%

As mentioned above, the initial humidity conditions before applying any RH regulations at room temperature were recorded to be 34%. By converting the moister conditions to absolutely dry (0% RH), the samples responded to decrease the required stress. However, increasing the humidity to 50% made the material stiffer, requiring more stress to be compressed. The results were similar for more than four high strain rates; however, we selected to show only two levels for each temperature for spacing-saving reasons.

In addition, when the temperature was increased to 60°C, the humidity in the environment was measured to be 0% RH considering absolute dry conditions. We repeated the experimental procedure for increased humidity levels at 34% RH and 50% RH to record and compare equivalent conditions for the two temperatures. The neoprene's response to this change differed from the corresponding behaviour recorded at 23°C temperature. Adding more humidity to the environment gradually degraded the samples' mechanical behaviour. As observed in Figure 3, the compressive stress level decreased with increasing humidity.

Finally, comparing Figure 2(b) to 3(a) for equivalent strain rate levels (270 s^−1^), it is evident that the rubber reduced its stiffness with increasing temperature regardless of the humidity conditions. This observation is similar for all strain rate levels reached in this study.

Consequently, unfilled neoprene is sensitive to elevated temperature, an assumption already known through the extended literature mentioned above. The quantitative changes (in %) of required compressive stress when increasing RH are presented in Table 1. These results prove that unfilled neoprene is highly affected by changing the moister, specifically from absolute dry conditions to increased RH percentage, where the required stress increases. This response is less evident (but still apparent) when the temperature increases, leading to diminishing stress levels. The results follow the results presented in other studies regarding the neoprene's response to temperature only.^3,6,7^

### Reinforced neoprene with material layers

**Figure 4.**
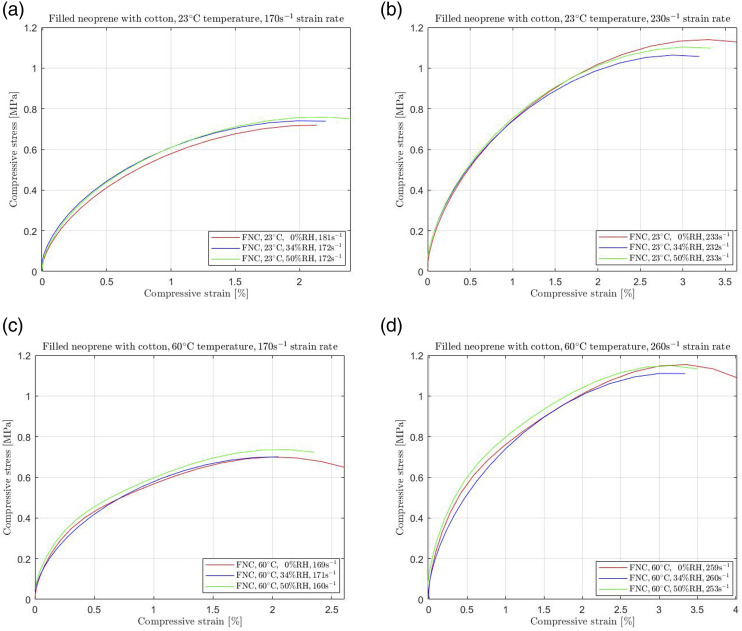
Filled neoprene with cotton fabric (FNC) subjected to (a) 170 s^−1^ and (b) 230 s^−1^ high strain rate compression at 23°C and (c) 170 s^−1^ and (d) 260 s^−1^ high strain rate compression at 60°C temperature.

A commonly used layer for reinforcing neoprene is cotton, which increases the duration of the mechanical properties by increasing its stiffness. In our investigation, we subjected filled neoprene with an intermediate cotton layer (FNC) to different humidity conditions and recorded its sensitivity. The procedure was performed not only at 23°C but at the elevated temperature of 60°C. Figure 4 shows that temperature and humidity changes have almost no impact on FNC samples' mechanical behaviour.

**Figure 5.**
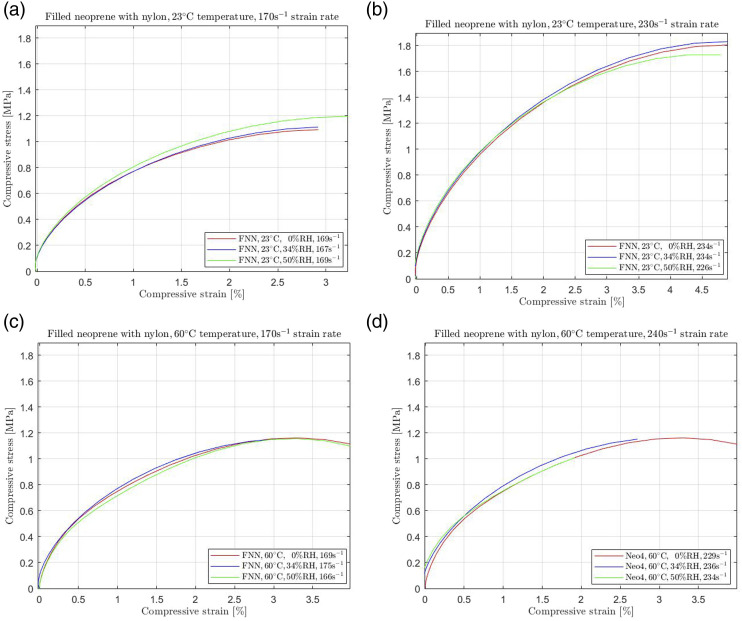
Filled neoprene with nylon (FNN) subjected to (a) 170s^−1^ and (b) 230s^−1^ high strain rate compression at 23°C and (c) 170s^−1^ and (d) 230s^−1^ high strain rate compression at 60°C temperature.

To verify the consistency of this behaviour for other filled neoprene rubbers, we repeated the same procedure on rubbers reinforced with nylon layers (FNN). Since nylon enhances the duration of the mechanical properties by increasing its stiffness, it was expected to record increased stress requirements for compressing the samples when exposed to high strain rate compression. Figure 5 shows the corresponding results at 23°C and 60°C temperatures.

Figures 4 and 5 show that the mechanical behaviour changes when neoprene is reinforced with an extra layer of enhanced material. Rubbers are becoming stiffer with intermediate layers, namely, requiring more stress to deform at a desired strain level. Depending on the filament material, the difference between unfilled and reinforced neoprene is significant. As shown in Figures 4 and 5, filled neoprene was recorded to exhibit resistance to increasing temperature and humidity; the stress-strain curves coincide for any strain rate.

Table 1 presents the quantitative changes in the required stress to compress the samples compared to the absolute dry conditions for each temperature. To ensure comparability, we selected each temperature's absolute dry (0% RH) environment as the reference condition (REF). These results corroborate the conclusions drawn from the preceding figures. Specifically, our investigation highlights significant disparities in the responses of unfilled neoprene and filled neoprene to temperature and humidity variations. UFN exhibits high sensitivity to these environmental factors, with stress decreasing as temperature increases and increasing stiffness with rising humidity levels at room temperature. However, elevated temperature combined with increased humidity leads to a reduction in the stiffness of UFN.^5,12,27^

In contrast, neoprene filled with nylon (FNN) and neoprene filled with cotton (NFC) display remarkable resilience to changes in temperature and humidity. FNN, in particular, showed an almost negligible effect, with less than 2% stress changes observed at any increased humidity level for both temperature settings. In addition, NFC also exhibited minimal effects from temperature rises and humidity changes, resulting in no more than a 5% alteration in their required stress. These results underscore the critical impact of temperature and humidity on unfilled neoprene while highlighting the stability and robustness of filled neoprene formulations, particularly those reinforced with nylon or cotton.

Consequently, the negligible impact of environmental changes on the mechanical behavior of filled rubbers is attributed to the intermediate cotton fabric or nylon layer, which acts as a “protection” against environmental variations. This striking contrast to the behaviour of unfilled neoprene is evident, with significant deviations of over 30% and 60% occurring at 34% RH and 50% RH, respectively, under room temperature conditions. Remarkably, even at 60°C, the response shows observable yet gradual alterations, with a stress reduction of 15% and 20% at 34% RH and 50% RH, respectively. Incorporating material layers in elastomers enhances their mechanical behavior, demonstrating that filled rubbers are effectively “protected” from environmental regulations, including humidity changes. Our study's findings align with similar investigations, indicating that unfilled rubbers are excessively affected by temperature changes.^3,7,23^ These variations in behaviour between the two filled rubbers are attributed to the different reinforcing materials used—specifically, samples that remain stiffer than cotton, even when exposed to temperature increases, irrespective of humidity levels.

## Conclusions

Elastomers exhibit strain-rate-dependent behavior in both quasistatic deformations and high strain rates where inertia significantly affects their mechanical behavior. Additionally, environmental changes can impact their stress response to deformation. The presence of material layers within neoprene samples can delay or eliminate the temperature effect but cannot block the impact of increasing high strain rates. Similarly, different humidity levels at room and elevated temperatures do not affect the mechanical behaviour of filled samples compared to pure neoprene. Unfilled neoprene experiences reduced stress values with increased humidity percentage, indicating a softening effect during compression. In contrast, neoprene rubbers filled with polyester/cotton or nylon resist changes in their mechanical response under various temperature and humidity conditions. The layer in the middle of the filled samples protects the materials from potential stress lapses observed in unfilled neoprene. Despite significant behavior changes under high strain rate compression, increasing humidity has no impact on the stress levels of filled rubbers.

In conclusion, our investigation provides valuable insights into the mechanical responses of unfilled neoprene (UFN) and filled neoprene under varying temperature and humidity conditions. The results demonstrate UFN's high sensitivity to environmental changes, while filled rubbers exhibit remarkable resilience, particularly those with nylon or cotton reinforcement. An intermediate layer acts as a “protective barrier,” enhancing the stability and robustness of filled rubbers. These findings have important implications for practical applications, emphasizing the significance of material selection and reinforcing layers in elastomers. The study highlights the advantages of using filled rubbers in environments subject to temperature and humidity variations, contributing valuable information to the field of rubber materials and aiding in developing more resilient and reliable products in diverse industrial and engineering applications.

## References

[1] Bridgewater E . Neoprene, the chloroprene rubber. Ind Eng Chem 1940; 32(9): 1155–1156.

[2] Trivedi A Siviour C . A simple rate–temperature dependent hyperelastic model applied. Journal of Dynamic Behavior of Materials 2020; 6: 336–347.

[3] Gkouti E Yenigun B Czekanski A . Transient effects of applying and removing strain on the mechanical behavior of rubber. Materials 2020; 13(19): 4333. 33003563 10.3390/ma13194333 PMC7579106

[4] Zakaria S Yu L Kofod G , et al. The influence of static pre-stretching on the mechanical ageing of filled silicone rubbers for dielectric elastomer applications. Mater Today Commun 2015; 4: 204–213.

[5] Rey T Chagnon G Le Cam J , et al. Influence of the temperature on the mechanical behaviour of filled and unfilled silicone rubbers. Polym Test 2013; 32(3): 492–501.

[6] Celina M Wise J Ottesen D , et al. Correlation of chemical and mechanical property changes during oxidative degradation of neoprene. Polym Degrad Stabil 2000; 68(2): 171–184.

[7] Bouaziz R Truffault L Borisov R , et al. Elastic properties of polychloroprene rubbers in tension and compression during ageing. Polymers 2020; 12(10): 2354. 33066496 10.3390/polym12102354 PMC7602244

[8] Roland C . Mechanical behavior of rubber at high strain rates. Rubber Chem Technol 2006; 79(3): 429–459.

[9] Shim J Mohr D . Using split Hopkinson pressure bars to perform large strain compression. Int J Impact Eng 2009; 36: 1116–1127.

[10] Iqbal N Tripathi M Parthasarathy S , et al. Polyurea coatings for enhanced blast-mitigation: a review. RSC advances 2016; 6(111): 109706–109717.

[11] Trivedi A Whybrow R Muhr A , et al. Experimentally characterising the temperature and rate dependent behaviour of unfilled, and glass microsphere filled, natural rubber. Polymer 2023; 270: 125773.

[12] Gkouti E Chaudhry M Yenigun B , et al. High-Strain-Rate Compression of Elastomers Subjected to Temperature and Humidity Conditions. Materials 2022; 15(22): 7931. 36431417 10.3390/ma15227931 PMC9698547

[13] Chaudhry M Czekanski A . Evaluating FDM process parameter sensitive mechanical performance of elastomers at various strain rates of loading. Materials 2020; 13(14): 3202. 32708369 10.3390/ma13143202 PMC7412201

[14] Fan J Weerheijm J Sluys L . High-strain-rate tensile mechanical response of a polyurethane elastomeric material. Polymer 2015; 65: 72–80.

[15] Conant F Hall G Lyons W . Equivalent effects of time and temperature in the shear creep and recovery of elastomers. J Appl Phys 1950; 21: 499.

[16] Chaudhry M Czekanski A . FE analysis of critical testing parameters in Kolsky bar experiments for elastomers at high strain rate. Materials 2019; 12(23): 3817. 31757077 10.3390/ma12233817 PMC6926648

[17] Raman S Ngo T Lu J , et al. Experimental investigation on the tensile behavior of polyurea at high strain rates. Mater Des 2013; 50: 124–129.

[18] Song B Chen W . Dynamic stress equilibration in split Hopkinson pressure bar tests on soft materials. Exp Mech 2004; 44: 300–312.

[19] Chen W Song B . Split Hopkinson (Kolsky) bar: design, testing and applications. Springer Science & Business Media, 2010.

[20] Chen W . Experimental methods for characterizing dynamic response of soft materials. Journal of Dynamic Behavior of Materials 2016; 2: 2–14.

[21] Siviour C Jordan J . High strain rate mechanics of polymers: a review. Journal of Dynamic Behavior of Materials 2016; 2: 15–32.

[22] Gama B Lopatnikov S Gillespie J Jr . Hopkinson bar experimental technique: a critical review. Appl Mech Rev 2004; 57(4): 223–250.

[23] Chang H Wan Z Chen X , et al. Temperature and humidity effect on aging of silicone rubbers as sealing materials for proton exchange membrane fuel cell applications. Appl Therm Eng 2016; 104: 472–478.

[24] Chouhan H Bhalla N Bhatnagar N . High strain rate performance of UHMWPE composites: Effect of moisture ingress and egress. Mater Today Commun 2021; 26: 101709.

[25] Zhang L Yao X Zang S , et al. Temperature and strain rate dependent tensile behavior of a transparent polyurethane interlayer. Mater Des 2015; 65: 1181–1188.

[26] Shit S Shah P . A review on silicone rubber. National academy science letters 2013; 36(4): 355–365.

[27] Chou H Huang J . Effects of ultraviolet irradiation on the static and dynamic properties of neoprene rubbers. J Appl Polym Sci 2008; 110(5): 2907–2913.

